# Identification of pathological pathways centered on circRNA dysregulation associated with early pathogenic progression of Alzheimer’s disease

**DOI:** 10.1002/alz.089983

**Published:** 2025-01-03

**Authors:** Feng Wang, Yangping Li, Huifeng Shen, Yue Feng, Bing Yao

**Affiliations:** ^1^ Emory University School of Medicine, Atlanta, GA USA

## Abstract

**Background:**

Circular RNAs (circRNAs) play multifaceted roles to precisely control expression of broad gene networks. These highly stable molecules are often accumulated in the mammalian brain and thought to serve as “memory molecules” that govern the long process of aging. Mounting evidence demonstrated circRNA dysregulation in the postmortem brains of Alzheimer’s disease (AD). However, whether and how circRNAs dysregulation underlies AD disease progression remain unexplored.

**Methods:**

Poly(A)‐tailing/RNase R digestion was coupled with a recently published computational algorithm called CARP to examine circRNA landscapes alteration and identify functional circRNA‐pathways progressively dysregulated in the cerebral cortex of the 5xFAD mouse model of AD between 5‐ and 7‐month, a critical window for the transition of starting pathology to full manifestation. We have also employed comprehensive biochemical approaches to identify circRNA interactome, as well as short hairpin RNA‐based genetic approaches to manipulate circRNAs and their key regulators to pinpoint their mechanistic contributions to AD.

**Results:**

We discovered genome‐wide alterations of brain circRNAs within the above age window critical for AD progression. Further analysis revealed co‐operation of distinct circRNAs and age‐dependent switch of circRNA isoforms derived from internal alternative splicing and alternative back splicing, which offered new means for regulating miRNA‐mRNA pathways in the 5xFAD cortex. In particular, we focused on conserved circRNAs that were progressively downregulated with increased severity of dementia in human AD brains. During circRNA biogenesis, we identified several previously established AD‐affected splicing factors, such as hnRNPL, regulate the production of AD‐associated circRNAs. Mechanistically, we found these circRNAs contained binding sites for many miRNAs and demonstrated its activity in sponging several AD‐relevant miRNAs to regulate their target mRNAs indicated in AD pathogenesis. Furthermore, we identified multiple circRNA‐interacting RNA binding proteins associated with AD, including the cleavage and polyadenylation factor 6 (CPSF6). In fact, altered polyadenylation efficiency was observed, which results in aberrant upregulation of many known CPSF6 target mRNAs indicated in AD pathogenesis.

**Conclusions:**

Together, our results unveiled pathological brain circRNA landscape alterations within a critical window for phenotypic progression in a mouse model of AD and identified novel molecular mechanisms underlying dysregulation of conserved circRNA pathways that contribute to AD pathogenesis.

